# NK-cell cytotoxicity toward pluripotent stem cells and their neural progeny: impacts of activating and inhibitory receptors and KIR/HLA mismatch

**DOI:** 10.1093/stmcls/sxae083

**Published:** 2024-12-21

**Authors:** Camilla Henden, Hege B Fjerdingstad, Elisabeth G Bjørnsen, Lavanya Thiruchelvam-Kyle, Michael R Daws, Marit Inngjerdingen, Joel C Glover, Erik Dissen

**Affiliations:** Department of Molecular Medicine, Institute of Basic Medical Sciences, University of Oslo, N-0317 Oslo, Norway; Department of Molecular Medicine, Institute of Basic Medical Sciences, University of Oslo, N-0317 Oslo, Norway; Norwegian Center for Stem Cell Research, Department of Immunology and Transfusion Medicine, Oslo University Hospital, N-0317 Oslo, Norway; Department of Molecular Medicine, Institute of Basic Medical Sciences, University of Oslo, N-0317 Oslo, Norway; Department of Molecular Medicine, Institute of Basic Medical Sciences, University of Oslo, N-0317 Oslo, Norway; Department of Molecular Medicine, Institute of Basic Medical Sciences, University of Oslo, N-0317 Oslo, Norway; Department of Pharmacology, Institute of Clinical Medicine, University of Oslo, N-0317 Oslo, Norway; Department of Molecular Medicine, Institute of Basic Medical Sciences, University of Oslo, N-0317 Oslo, Norway; Norwegian Center for Stem Cell Research, Department of Immunology and Transfusion Medicine, Oslo University Hospital, N-0317 Oslo, Norway; Department of Molecular Medicine, Institute of Basic Medical Sciences, University of Oslo, N-0317 Oslo, Norway

## Abstract

Pluripotent stem cells provide opportunities for treating injuries and previously incurable diseases. A major concern is the immunogenicity of stem cells and their progeny. Here, we have dissected the molecular mechanisms that allow natural killer (NK) cells to respond to human pluripotent stem cells, investigating a wide selection of activating and inhibitory NK-cell receptors and their ligands. Reporter cells expressing the activating receptor NKG2D responded strongly to embryonic stem (ES) cell lines and induced pluripotent stem (iPS) cell lines, whereas reporter cells expressing the activating receptors NKp30, NKp46, KIR2DS1, KIR2DS2, and KIR2DS4 did not respond. Human ES and iPS cells invariably expressed several ligands for NKG2D. Expression of HLA-C and HLA-E was lacking or low, insufficient to trigger reporter cells expressing the inhibitory receptors KIR2DL1, -2DL2, or -2DL3. Similar results were obtained for the pluripotent embryonic carcinoma cell lines NTERA-2 and 2102Ep, and also iPS-cell-derived neural progenitor cells. Importantly, neural progenitor cells and iPS-cell-derived motoneurons also expressed B7H6, the ligand for the activating receptor NKp30. In line with these observations, IL-2-stimulated NK cells showed robust cytotoxic responses to ES and iPS cells as well as to iPS-cell-derived motoneurons. No significant differences in cytotoxicity levels were observed between KIR/HLA matched and mismatched combinations of NK cells and pluripotent targets. Together, these data indicate that pluripotent stem cells and their neural progeny are targets for NK-cell killing both by failing to sufficiently express ligands for inhibitory receptors and by expression of ligands for activating receptors.

## Graphical Abstract

**Graphical Abstract:**
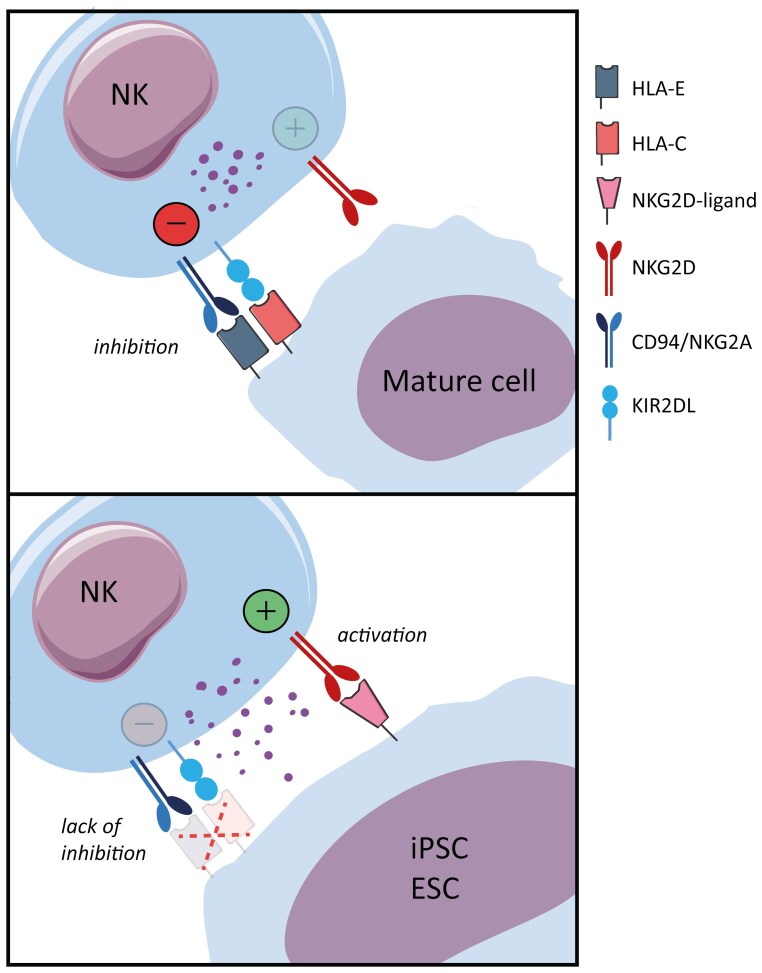

**Significance statement**

Successful stem-cell-based transplantation depends on immunogenicity. In this paper, comprehensive molecular and functional dissection of individual NK-cell receptors and their stem cell ligands revealed an NK-cell-susceptible surface expression phenotype consistently associated with pluripotency. This was also seen with neural progenitor cells and motoneurons derived from induced pluripotent stem cells. These data provide a basis for approaches to optimize the expression of ligands for NK-cell receptors to reduce immunogenicity of stem cell progeny.

## Introduction

Pluripotent stem cells can be differentiated into cells from all 3 embryonic germ layers. Pluripotency is normally restricted to embryonic stem (ES) cells of the blastocyst inner cell mass but can also be a feature of germ-cell-derived ECs.^1^ In vitro, pluripotency is a feature of ES cell lines and of induced pluripotent stem (iPS) cells generated by epigenetic reprogramming of somatic cells.^2^ Techniques to differentiate diverse cell types from pluripotent stem cells in vitro are developing rapidly. One example of a well-characterized differentiation pathway is the generation of neurons from pluripotent stem cells, via neural progenitor cells (NPCs).^3^

These advances in pluripotent stem cell research are facilitating the development of therapeutic applications, including implantation of stem cell derivatives to restore function in damaged tissues (eg, Parkinson’s disease, spinal cord injury, retinopathies, and heart failure),^4-7^ and the in vitro generation of whole tissues and organs from iPS cells.^8,9^

Several concerns have arisen regarding the transplantation of either pluripotent stem cells, more differentiated progenitor stages, or fully differentiated cells. These include not only immune rejection of transplanted cells but also the spontaneous development of transplant-derived tumors.^10,11^ If unchecked by immunosurveillance mechanisms, pluripotent stem cells can give rise to teratomas and possibly other tumors in vivo.^12,13^ Differentiated cells may also spontaneously regain pluripotency, as reported for iPS cells reprogrammed by retrovirally mediated integration of exogenous pluripotency genes.^14^

Considerable attention has been given to manipulating stem cells to prevent rejection of their differentiated progeny by the immune system.^15,16^ Although in the context of clinical implantation autologous iPS cells would be preferable due to HLA identity, the time and resources required to generate iPS cells from a patient can greatly restrict their utility as a treatment strategy. To overcome this hurdle, banks of iPS cells derived from a limited number of HLA homozygous donors are being established.^17,18^ However, while implantation of such partially HLA-matched stem cells would not provoke the T-cell responses seen in allogeneic transplant rejection, they could still be targets for NK cells in the recipient.^19^

Natural killer (NK) cells belong to the innate immune system and can spontaneously recognize and kill cancer cells and infected cells.^20-22^ NK cells kill target cells through the release of cytotoxic granules containing perforin and granzymes, or alternatively, by interaction with death receptors expressed on target cells. By releasing cytokines such as IFN-γ, GM-CSF, and TNF-α, NK cells are also important modulators of adaptive immune responses.^20,21^ NK cells express a wide repertoire of clonally distributed activating and inhibitory receptors. The effector functions of NK cells are thus controlled by a balance of activating and inhibitory signals in the immunological synapse formed in contact with the target cell.^23^

In humans, the killer cell immunoglobulin-like receptor (KIR) family consists of both activating and inhibitory family members. The inhibitory receptors KIR2DL1, -2, and -3 recognize allelic versions of HLA-C, whereas KIR3DL1 and -2 specifically bind to allelic variants of HLA-B, HLA-A, or HLA-F.^24-26^ Target cells that fail to express sufficient amounts of class I HLA ligands for these inhibitory receptors are killed by NK cells. In contrast, the ligand specificities of the activating KIR family members are still poorly understood.^26^ Some recent reports indicate that activating KIR2DS receptors display peptide specificity and can bind to certain HLA-C alleles presenting evolutionarily conserved pathogen-derived peptides.^27,28^ Binding to surface ligands on cancer cells has also been reported.^29^

CD94/NKG2 is a second family of activating and inhibitory heterodimeric receptors.^23^ CD94/NKG2A is an inhibitory receptor that binds MHC class I-derived leader peptides presented by HLA-E,^30^ whereas CD94/NKG2C is an activating receptor reported to recognize viral peptides presented by HLA-E.^31,32^

In addition to the receptor families with both inhibitory and activating members, NK cells express a number of individual activating receptors, including NKG2D, NKp30, and NKp46.^20^ NKG2D binds to a set of MHC class I-like ligands; MICA and -B and ULBP1 through -6.^33,34^ Expression of these ligands is regulated by different mechanisms that can be induced by virus infection, DNA damage, and oxidative stress, among others.^34^ NKp30 binds to the B7 family member B7H6, which is commonly expressed at the surface of cancer cells.^35,36^ B7H6 expression has been reported on myeloid cell subsets in sepsis^37^ and in T cells following T-cell receptor crosslinking,^38^ but there is at present little evidence to support B7H6 expression by healthy non-immune cells. Additionally, several alternative ligands have been reported for NKp30.^39^ NKp46 is another activating receptor, expressed by all NK cells. Several unrelated ligands have been reported, including ecto-calreticulin,^40^ many of which remain to be robustly confirmed.^39^ Previous reports have found that mouse and human pluripotent stem cells can express ligands for NKG2D,^41,42^ and express reduced levels of MHC class I molecules.^43,44^ Although conflicting results have been reported,^45^ NK cells have been shown to be able to kill pluripotent stem cells in vitro^41,44,46-48^ and to prevent teratoma formation in mice transplanted with pluripotent stem cells.^46,49^ Although the molecular basis for NK-cell responses toward pluripotent stem cells has been partly explained, NK-cell-mediated killing of human ES and iPS cells has thus far been demonstrated without attention to the impact of KIR/HLA mismatch between stem cell targets and NK-cell donors. Previous reports found ES cells and iPS cells to be susceptible to killing by syngeneic NK cells, suggesting that allorecognition is not necessary.^44,46,48^ To investigate further the possibility that reported cases of NK-cell killing of pluripotent human stem cells is partly due to alloreactivity by missing-self recognition mechanisms, rather than a specific targeting of pluripotent stem cells, we have evaluated NK-cell susceptibility in carefully KIR- and MHC-genotyped allogeneic combinations. Moreover, recognition of pluripotent stem cells by activating KIR, NKp30, and NKp46 receptors has not been thoroughly investigated. In addition, although reduced expression of MHC class I has been reported in mouse and human, surface expression of HLA-C on human pluripotent stem cells has been less precisely studied.

Here, using reporter cells for a broad panel of activating and inhibitory NK-cell receptors, we have functionally dissected receptor-ligand interactions that regulate NK-cell cytotoxicity toward human pluripotent cells, including ES, iPS, and human EC cell lines, as well as a well-characterized iPS-derived neural lineage including neural progenitor cells and motoneurons (MNs).

## Materials and methods

### ES cell lines

Human ES cell lines HS360 (hPSCreg ID Kle009-A), HS401 (ID Kle019-A), and HS429 (ID Kle024-A) were kindly provided by Outi Hovatta, Karolinska Institute. The human ES cell line H9 (WA09, ID WAe009-A) was obtained from WiCell. The ES cells were cultured in Matrigel (Corning) coated wells in Essential 8 medium (Gibco) according to standard protocols.

### Generation of iPS cells

Generation of iPS cells was performed at the National Core Facility for Human Pluripotent Stem Cells at the Norwegian Center for Stem Cell research, in compliance with the Regional Committee for Medical and Health Research Ethics 2011/2617. Fibroblasts were obtained from skin biopsies from 4 healthy donors using conventional methods after participants gave informed consent. Reprogramming was achieved using the CytoTune iPS-Sendai Reprogramming kit (Thermo Fisher). The resultant iPS cells were registered as lines NCS033, NCS001, NCS002, and NCS004 in the National Core Facility for Human Pluripotent Stem Cells biobank.

### ES and iPS cell characterization

Pluripotency of ES and iPS cells was confirmed both by immunofluorescence and quantitative RT-PCR (qPCR). For immunofluorescence, cells were fixed with 4% paraformaldehyde (Sigma-Aldrich) and incubated with blocking buffer (PBS containing 20% normal goat serum, 0.1% Triton X-100 (Sigma-Aldrich)) for 30 minutes at room temperature. Following permeabilization in 0.1% Triton, the cells were stained with primary antibodies against the pluripotency markers Nanog (clone 7F7.1; 1:400; Millipore), Oct4, and SOX2 (cat. no. 090023 and 090024, respectively; 1:100; StemGent) overnight at 4 °C, washed, incubated for 1 hour at room temperature with Cy2-conjugated or Cy3-conjugated secondary antibodies (1:400; Jackson ImmunoResearch) followed by counterstaining of nuclei with DAPI. Microphotographs were taken with an LSM700 confocal fluorescence microscope and analyzed with ZEN software (Zeiss). For qPCR analysis, total RNA was extracted using TRIzol reagent (Thermo Fisher) and 1st strand cDNA was synthesized using the high capacity cDNA RT kit (Thermo Fisher) following the manufacturer’s recommendations. qPCR analysis was performed with gene-specific primers and TaqMan probes (FAM-MGB) according to the manufacturer’s protocol. β-actin (ACTB) was used as an internal control, and relative expression was calculated as 2^−ΔΔCt^. All primers and probes assays were from Thermo Fisher with the following references: β-actin (ACTB) 4333762F; Oct4 (POU5F1) 4351370-Hs00999634_gH; Nanog (NANOG) 4351370-Hs04260366_g1, and SOX2 (SOX2) 4351370-Hs01053049_s1.

### Differentiation of NPCs

#### Differentiation of NPCs was accomplished using dual-SMAD inhibition

Generation of NPCs from iPS cells involved 3 steps: neuralization (phase I), caudalization (phase II), and ventralization (phase III). iPS cells were plated on reduced-growth factor Matrigel (Corning). Phase I takes typically 5-7 days using advanced DMEM/F12, 1% Glutamax, 1% N2 supplement, 1% penicillin/streptomycin, 10 µM SB431542, 100 nM LDN193189, and 2 µM XAV939. Neural rosettes were transferred using rosette selection reagent (Stem Cell Technologies) to new laminin-coated plates and cultured in phase II medium consisting of advanced DMEM/F12, 1% Glutamax, 1% P/S, 1% N2 supplement, 0.4% B27 supplement with retinoic acid (RA), and 2.5 ng/mL bFGF (PeproTech). After 4-5 days, cells were transferred using accutase to new laminin-coated plates and cultured in phase III medium consisting of advanced DMEM/F12, 1% Glutamax, 1% penicillin/streptomycin, 1% N2 supplement, 0,1% B27 + RA, and 10 ng/mL bFGF. Cells were passaged using accutase on laminin-coated plates for 5-6 passages and maintained on reduced growth factor Matrigel-coated plates typically until passage 10 for further experiments.^50^ Phase II and III cultures were in reduced (5%) oxygen atmosphere. The generated NPCs were named NPC033 (generated from NCS033), NPC001 (from NCS001), NPC002 (from NCS002), and NPC004 (from NCS004). qPCR was used to confirm expression of nestin (NES) (TaqMan assay 4331182-Hs04187831_g1, Thermo Fisher) and loss of Oct4 (POU5F1), see above.

### Differentiation of NPCs to MNs

Differentiation of NPCs to motoneuron precursors (pMNs) was induced by culture in phase III medium for 2 passages. After 4 days cells were transferred using accutase to new reduced growth factor Matrigel-coated-plates in medium consisting of advanced DMEM/F12, 1% Glutamax, 1% penicillin/streptomycin, 1% N2 supplement, 0.1% B27 + RA, 0.1 µM RA, 2 µM purmorphamine, and 5 ng/mL bFGF and cultured for 7-10 days. Differentiation of pMNs to MNs started by transferring pMNs using accutase to 12-well plates containing coverslips pre-coated with 15 µg/mL polytornithine and 5 µg/mL laminin, and culturing in advanced DMEM/F12, 0.5% Glutamax, 1% penicillin/streptomycin, 0.5% N2 supplement, 0.2% B27 + RA, 5 µg/ml BDNF, and 5 µg/mL GDNF. Cells were cultivated for 50 days with medium change every fourth day. TaqMan qPCR (ThermoFisher) was used to confirm expression of the neuronal markers with the following TaqMan assays (ThermoFisher): NeuN (RBFOX3) 4331182-Hs01370654_m1); TuJ1 (Class III β-tubulin, TUBB3) 4331182-Hs00801390_s1; synaptotagmin (SYT1) 4331182-Hs00194572_m1; and MAP-2 (MAP2) 4331182-Hs00258900_m1, as described above. Parallel electrophysiological characterization was performed using conventional patch clamp methods in current-clamp and voltage-clamp modes.

### Flow cytometry

Purified monoclonal antibodies against the following antigens were used: HLA-A, -B, and -C (clone B9.12.1; Beckman Coulter; 2 µg/mL), HLA-C and -E (clone DT9; Merck Millipore; 4 µg/mL), HLA-C (clone, L-31, MediaPharma; 5 µg/mL), HLA-B and -C including open conformers (clone HC10; 4 µg/mL), HLA-E (clone 3D12, eBioscience; 8 µg/mL), HLA-F (clone 3D11, BioLegend; 5 µg/mL), HLA-G (clone 4H84, BD Biosciences; 8 µg/mL), B7H6 (clone 875001, R&D Systems; 2 µg/mL), ULBP4 (R&D Systems; 2 µg/mL), and ULBP2/-5/-6 (R&D Systems; 2 µg/mL). MICA (clone AMO1; 2 µg/mL), MICB (clone BMO2; 2 µg/mL), ULBP1 (clone AUMO3; 2 µg/mL), ULBP2 (clone BUMO2; 2 µg/mL), and ULBP3 (clone CUMO3; 2 µg/mL) were a kind gift from Alexander Steinle, Goethe University, Frankfurt. Cells were washed with PBS with 2 % FBS and 10 mM NaN_3_ and incubated in 30-50 µL PBS/FBS/NaN_3_ with primary antibody for 30 minutes on ice. Cells were then washed and incubated in 30-50 µL PBS/FBS/NaN_3_ with a secondary goat anti-mouse IgG polyclonal antibody conjugated with Alexa Fluor 647 (Molecular Probes) for 30 minutes on ice. Finally, the cells were washed in PBS with 10 mM NaN_3_ and run on a flow cytometer (FACSCanto II or FACSCalibur, BD Biosciences). Flow cytometry data analysis was performed with CellQuest, FACS Diva (both BD Biosciences), and FlowJo software.

### Chimeric receptor reporter cell lines

To generate EGFP-expressing reporter cell lines for human NKG2D, an expression construct encoding a chimeric fusion protein consisting of an N-terminal FLAG epitope tag (DYKDDDDK), the extracellular region of human NKG2D, the transmembrane region of human CD8, and the cytoplasmic tail of mouse CD3ζ, was generated in the pBSRɑ-EN vector^51^ and verified by sequencing. The plasmid was then linearized and used to transfect BWN3G cells^52^ by electroporation. BWN3G cells (3 × 10^6^) were resuspended in 400 µL of cRPMI containing 20 µg of plasmid, and electroporated in 2 mm cuvettes (120 V, 960 µF, Genepulser II; Bio-Rad Laboratories). After 24 hours, transfected cells were seeded in 96-well plates at between 10 000 and 1000 cells per well in selection medium (cRPMI supplemented with 1.6 mg/mL geneticin (G-418 disulfate; Thermo Fisher Scientific)) and 1 mg/mL hygromycin (Invitrogen). Clones with bright NKG2D surface expression were identified by flow cytometry (clone 5C6, eBioscience; 2 µg/mL) and tested for EGFP expression following receptor crosslinking: 96-well culture plates were coated with secondary Ab (goat polyclonal anti-mouse IgG; Jackson ImmunoResearch; 10 µg/mL) in coating buffer (50 mM sodium carbonate buffer, pH 9.3) at 4 °C overnight, washed and blocked for 30 minutes at room temperature with PBS containing 10 mg/mL BSA (Sigma-Aldrich), then coated with anti-FLAG primary Ab (mAb clone M2, Sigma-Aldrich 10 µg/mL) at 4 °C overnight and washed with PBS. Finally, 5 × 10^4^ reporter cells were added per well in 100 µL of cRPMI and incubated overnight at 37 °C followed by flow cytometry analysis of EGFP expression. BWN3G reporter cell lines expressing human KIR2DS1, KIR2DS2, KIR2DS4, KIR2DL1, KIR2DL2, KIR2DL3, NKp30, or NKp46 were previously generated and selected in the same manner.^29,36^ Reporter and control target cell lines were cultured in RPMI 1640 supplemented with 1 mM sodium pyruvate, 1% antibiotic/antimycotic solution, and 10% FCS (Invitrogen), hereafter referred to as complete RPMI (cRPMI) at 37 °C in humidified air containing 5% CO_2_. Control cell lines used were HEK293T, K562, DU145, HCT15, MDA.MB.231, and 721.221 cells untransfected or stably transfected with HLA-C*03:04 or -C*15:03.

### Reporter cell assay

Reporter cell EGFP expression was analyzed by flow cytometry following co-incubation with stem cell or stem-cell-derived neural lineage targets. To avoid differentiation of pluripotent stem cells during the assay, they were seeded in a Matrigel-coated 96-well plate and cultured in E8 medium until 70-80% confluence was reached before the reporter cells were added. 5 × 10^4^ reporter cells were added to each well in 200 µL of E8 medium, and the cells were co-incubated for 6 hours at 37 °C. For assays with iPS-derived motoneurons, targets were maintained in 24-well plates in neuron medium as previously described and co-incubated with 3 × 10^5^ reporter cells for 6 hours at 37 °C. After co-incubation, cells were stained with an anti-HLA class I antibody (mAb W6/32^53^ conjugated to AlexaFluor 647, 4 µg/mL) to allow a specific analysis of EGFP expression in the reporter cells (mouse) gated away from the target stem cells (human).

### NK-cell cytotoxicity and IFN-γ assay

Peripheral blood mononuclear cells (PBMC) were separated from buffy coats by density gradient centrifugation using Lymphoprep (Axis-Shield), and cells were viably cryopreserved in 20% DMSO and 80% FBS. Buffy coats were obtained from healthy donors at the Blood Bank, Oslo University Hospital in compliance with the Regional Committee for Medical and Health Research Ethics (REK 20121452). The cells were thawed the day before the assay and incubated overnight at a concentration of 1 × 10^6^ cells/mL in cRPMI with or without human recombinant IL-2 (TECIN (teceleukin); Roche; 500 IU/mL). On the day of the assay, the PBMCs were washed and resuspended in target cell-optimized medium. For pluripotent stem cell targets 1 × 10^5^ PBMCs were added to 96-well plate wells containing stem cells in 100 µL E8 medium. The target stem cells had been seeded and cultured in the 96-well plate to reach 70-80% confluence on Matrigel in E8 medium prior to the assay. The NK-susceptible target cell line K562 was used as a positive control, and Matrigel-coated wells without target cells served as a negative control. The cells were stained with an anti-CD107a V450-conjugated mAb (clone H4A3, BD Biosciences; 5 µL/test) or isotype control immediately after the addition of the PBMCs to the stem cells. The cells were then co-incubated for a total of 4 hours at 37 °C. Brefeldin A (Sigma-Aldrich; 10 µg/mL) was added after 1 hour. Following the incubation, the cells were stained with the following antibody conjugates in 100 µL PBS with 2% FBS: FITC conjugated cocktail of anti-CD3, -CD14, -CD19 and -CD20 (anti-human lineage cocktail 3, BD Biosciences; 5 µl/test) and anti-CD56-APC (clone B159, BD Biosciences; 20 µL/test). Following surface staining, the cells were fixed in 100 µL of PBS with 2% paraformaldehyde for 10 minutes at RT and then permeabilized in PBS with 2% FBS and 0.5% saponin (Sigma-Aldrich) for 20 minutes at RT. Finally, the cells were stained with anti-IFNγ-PE (clone 45B3, BD Biosciences; 20 µL/test) or isotype control in 100 µL of PBS/2% FBS/0.5% saponin solution for 20 minutes on ice, washed, and analyzed by flow cytometry. For iPS-derived motoneuron targets, assays were performed in a similar manner in 6 well plates. Motoneurons were cultured in neuron medium as described, and 3 × 10^5^ PBMCs suspended in 300 µL neuron medium containing CD107a antibody (7.5 µL/well) or Ig control antibody (0.3 µL/well) were carefully added to each well. Cells were co-incubated for 140 minutes. After co-incubation, cells were stained as described for stem cell assays and analyzed by flow cytometry. For statistical analysis, one-way ANOVA was performed to study variability between the different PBMC donors in a group (unstimulated or IL-2 stimulated), whereas a paired Student’s *t*-test was performed to analyze the difference between groups of unstimulated and IL-2 stimulated donors.

### KIR and HLA genotyping

Genomic DNA from several stem cell lines and PBMCs was extracted by a proteinase K/phenol/chloroform protocol as previously described. KIR genotyping was performed by PCR with a sequence-specific primer (SSP) typing kit (Miltenyi Biotec) followed by agarose gel electrophoresis and visualization by ethidium bromide staining. HLA genotyping was performed similarly with 2 different SSP typing kits: KIR HLA Ligand-SSP and HLA-C low resolution-SSP, analyzed using SCORE software (Olerup). All iPS lines used in this study are derived from anonymous donors, who are protected from knowledge of incidental findings such as those obtained through the specific genotyping implemented here, according to the ISSCR Guidelines for stem cell research and clinical translation.

### B7H6 transcription

Total RNA from cell lines or primary cells was isolated using TRIzol reagent according to the manufacturer’s instructions (Life Technologies). First-strand cDNA synthesis was carried out using a modified M-MLV RNase H-reverse transcriptase (Superscript III, Invitrogen, manufacturer´s instructions) using 1 µg of total RNA in a 20 µL reaction. qPCR was performed in triplicates in 384-well plates by a standard TaqMan protocol with specific primers from neighboring exons for human B7H6 (NCR3LG1) (4331182-Hs02340611 m1, Thermo Fisher), GNB2L1, and GAPDH, respectively, using Platinum Quantitative PCR Supermix-UDG with ROX (Invitrogen) and a 7900HT thermal cycler (Applied Biosystems). The data were analyzed by the ΔΔCt method.

## Results

### Pluripotent stem cells express ligands for the activating NK-cell receptor NKG2D and activate NKG2D reporter cells

NK-cell reactivity toward human pluripotent stem cells has been reported previously. To investigate the underlying mechanism, we have investigated NK-cell recognition of human pluripotent stem cells at the molecular level, using NK-receptor reporter cells, This approach allows monitoring of receptor-ligand interaction in a physiological setting that requires cell-cell contact and the formation of an immunological synapse. Besides the benefit of a more physiological assay, the reporter cell approach allows the detection of NK-receptor ligand expression by stem cells regardless of whether the identity of the ligands is known beforehand.

**Figure 1.**
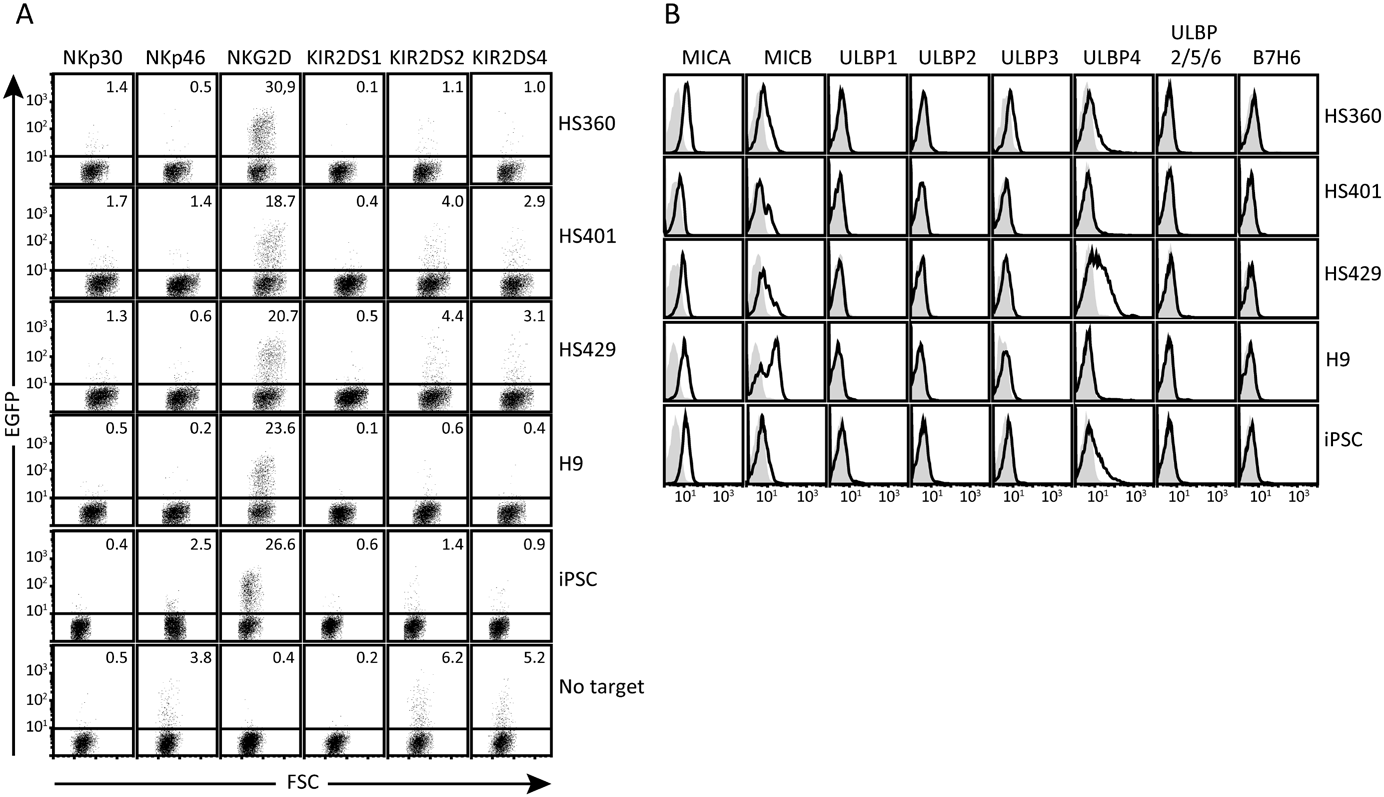
Expression of ligands for activating NK-cell receptors by human pluripotent stem cells. (A) Four human ES cell lines (HS360, HS401, HS429, and H9) and 1 human iPS cell line (NCS033) were co-incubated with reporter cells expressing chimaeric versions of the indicated activating NK-cell receptors in E8 medium for 6 hours. Reporter cell EGFP expression was measured by flow cytometry, gating on HLA class I negative cells to separate reporters (mouse) from targets (human). Percent EGFP+ reporter cells in the displayed experiments are indicated. The data shown are representative of at least 3 independent experiments. (B) Flow cytometry analysis of 4 human ES cell lines (HS360, HS401, HS429, and H9) and 1 iPS cell line (NCS033) using mAbs against ligands for NKG2D (MICA, MICB, ULBP1, ULBP2, ULBP3, ULBP4, ULBP5, and -6) and NKp30 (B7H6) (black lines) compared to control Ig (gray shading) are shown. The data shown are representative of at least 3 independent experiments.

Reporter cells expressing activating human NK cell receptors (NKp30, NKp46, NKG2D, KIR2DS1, KIR2DS2, or KIR2DS4) were assayed against 4 human ES cell lines (HS360, HS401, HS429, and H9) and 1 human iPS cell line (NCS033). Pluripotency was verified by immunofluorescence and quantitative RT-PCR, confirming the expression of the pluripotency markers Nanog, Oct4, and SOX2 in all 5 cell lines (Supplementary Figure S1). To maintain unaltered stem-cell phenotypes, the reporter cell assays were performed for short intervals (6 hours) in stem-cell medium (E8) using undisturbed human ES and iPS cells pre-incubated on Matrigel-coated plastic wells. In these assays, all the pluripotent stem cell targets induced activation of NKG2D reporters, whereas no activation was observed with reporters expressing NKp30, NKp46, KIR2DS1, -2DS2, or -2DS4 (Figure 1A and Supplementary Figure S2). In positive control experiments, all reporter cells responded to target cells known to express ligands or to crosslinking by incubation in culture wells precoated with antibody (Supplementary Figure S3). Background reactivity for each reporter cell was assessed by incubating reporter cells in wells without target cells (Figure 1A, bottom row) or with target cells lacking ligands for the different receptors (data not shown).

To further characterize the observed reactivity with NKG2D, we directly assessed pluripotent stem cells for surface expression of known ligands for this receptor by flow cytometry. All 4 of the ES cell lines expressed MICA and MICB to varying degrees. Surface expression of ULBP1, -2, -5, or -6 was not detected, but ULBP4 was expressed clearly by HS429 cells and weakly by HS360 cells. Three of the 4 ES cell lines weakly expressed ULBP3 (Figure 1B). The iPS cell line displayed a very similar phenotype to the ES cells but with a lower expression of MICB (Figure 1B). These observations together point to MICA, MICB, and ULBP4 as the ligands that account for the reactivity with NKG2D reporters. None of the tested stem cell lines expressed the NKp30 ligand B7H6, consistent with their inability to activate the NKp30 reporter cells.

### Pluripotent stem cells lack or express low levels of ligands for inhibitory NK receptors and fail to trigger inhibitory KIR reporter cells

**Figure 2.**
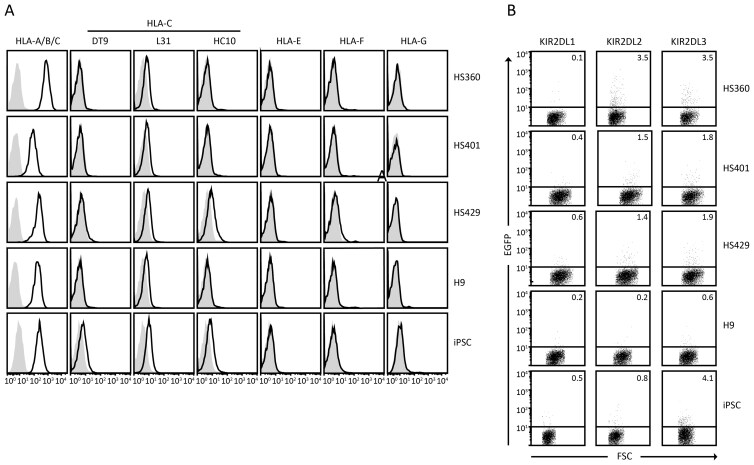
Surface expression of HLA class I molecules on human pluripotent stem cell lines. (A) Flow cytometry analysis of HLA class I surface expression on 4 human ES cell lines (HS360, HS401, HS429, and H9) and 1 human iPS cell line (NCS033). The 3 HLA-C reactive mAbs used (DT9, L31, and HC10) can also bind other HLA class I molecules besides HLA-C (as described in the Materials and methods section). Black lines represent specific mAb staining, control Ig staining is indicated by gray shading. (B) Flow cytometry analysis of EGFP expression by reporter cells expressing the indicated inhibitory receptors following 6 hours co-incubation with the indicated human pluripotent stem-cell targets. Reporter cell responses significantly above background were not detected. Percent EGFP+ reporter cells in the displayed experiments are indicated. The cells were gated on HLA class I negative cells to separate reporters (mouse) from targets (human). Data shown in (A) and (B) are representative of at least 3 independent experiments.

NK-cell cytotoxicity is regulated by a balance of simultaneous inputs from activating and inhibitory surface receptors. Accordingly, NK-cells can kill targets that fail to express ligands for inhibitory receptors such as CD94/NKG2A (ligand HLA-E) and inhibitory members of the KIR family (ligands HLA-C and some variants of HLA-A and -B). We therefore investigated the expression of ligands for inhibitory receptors on the same ES and iPS cell lines. Assessed by flow cytometry, HLA-E was not expressed by any of the tested pluripotent stem cell lines, indicating that they would not be protected from NK-cell killing via engagement of the inhibitory CD94/NKG2A receptor (Figure 2A). With regard to ligands for inhibitory KIR, a mAb broadly reacting with HLA-A, -B, and -C brightly stained all pluripotent stem cells tested (Figure 2A), at levels comparable with other normal, differentiated cell types (not shown). Three different mAbs against HLA-C, however, stained only at very low levels in the 4 ES cell lines and the iPS cell line (Figure 2A). Of the 3 HLA-C reactive mAbs used, L31 is HLA-C specific, whereas mAb DT9 binds to HLA-C and -E, and mAb HC10 binds to HLA-B and -C. L31 and HC10 can also bind to HLA-C in an open conformation, lacking peptide. Together, these data indicate that these pluripotent stem cells expressed high levels of HLA-A and/or HLA-B, but only very low levels of HLA-C, possibly restricted to open conformers that would not be expected to bind inhibitory KIR (Figure 2A). To investigate the ability of these stem cells to engage with inhibitory KIR receptors specific for HLA-C, they were co-incubated with reporter cells expressing KIR2DL1, -2DL2, or -2DL3. None of the stem cell lines induced significant responses by these reporters (Figure 2B), in agreement with the observed low levels of HLA-C expression. Finally, none of the pluripotent stem cell lines expressed HLA-F or -G (Figure 2A).

### Expression of NK-receptor ligands by EC cell lines with pluripotent characteristics

**Figure 3.**
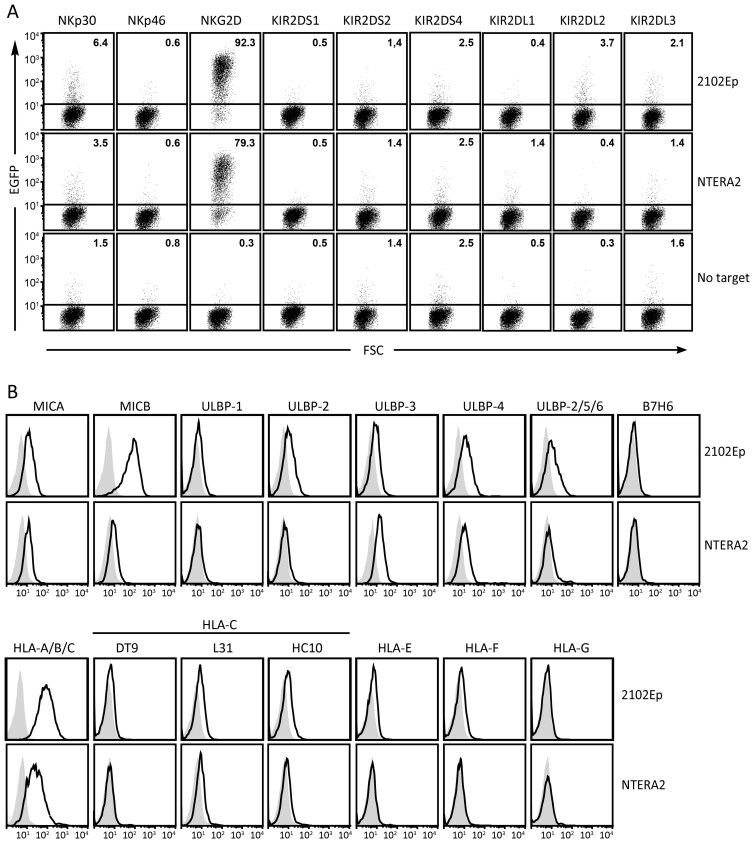
Reporter cell assays and flow cytometry analysis of ligand expression by human embryonic carcinoma cell lines. (A) Reporter cell assays using reporters expressing the indicated activating or inhibitory NK-cell receptors. The dot plots display EGFP expression by reporter cells after co-incubation with human embryocarcinoma cell lines 2102Ep or NTERA2. The cells were gated on HLA class I negative cells to separate reporter cells (mouse) from target cells (human). (B) Flow cytometry analysis of NK-receptor ligand expression by NTERA2 or 2102Ep cells. The mAbs DT9, L31, and HC10 react with different allelic forms and conformations of HLA-C. The data shown are representative of at least 3 independent experiments.

The human EC cell lines NTERA2 and 2102Ep, derived from germ cell cancers, share several features with human ES cells. They are considered to be pluripotent and can be differentiated into multiple lineages. In parallel with the above investigations of ES and iPS cells, we analyzed the capacity of these EC cell lines to engage reporter cells for activating as well as inhibitory NK-cell receptors. As for ES and iPS cells, no response was observed with reporters for the activating receptors NKp46, KIR2DS1, -2DS2, or -2DS4 (Figure 3A). In contrast, NKG2D reporters responded vigorously to both these targets (Figure 3A). On the inhibitory side, neither target efficiently triggered KIR2DL1, -2, and -3 reporters (Figure 3A). Assessed by flow cytometry, both NTERA2 and 2102Ep cells expressed several ligands for NKG2D, in accordance with the strong NKG2D reporter responses (Figure 3B). Despite the failure of NTERA2 and 2102Ep to efficiently trigger reporters for HLA-C-specific inhibitory KIR, weak staining of NTERA2 cells was observed with mAbs HC10 and L31. Both of these mAbs bind HLA-C in native as well as open conformation. Notably, mAb DT9, reactive with HLA-C and HLA-E, did not stain NTERA2. Together with the reporter cell data, these observations suggest that NTERA2 cells express low levels of HLA-C, mostly in open conformation that would most likely not bind to inhibitory KIR. Although weak responses were occasionally observed with NKp30 reporter cells, we could not detect significant levels of cell surface B7H6 using flow cytometry (Figure 3).

### Pluripotent stem cells are targets for cytotoxicity by KIR/HLA-matched NK cells

**Figure 4.**
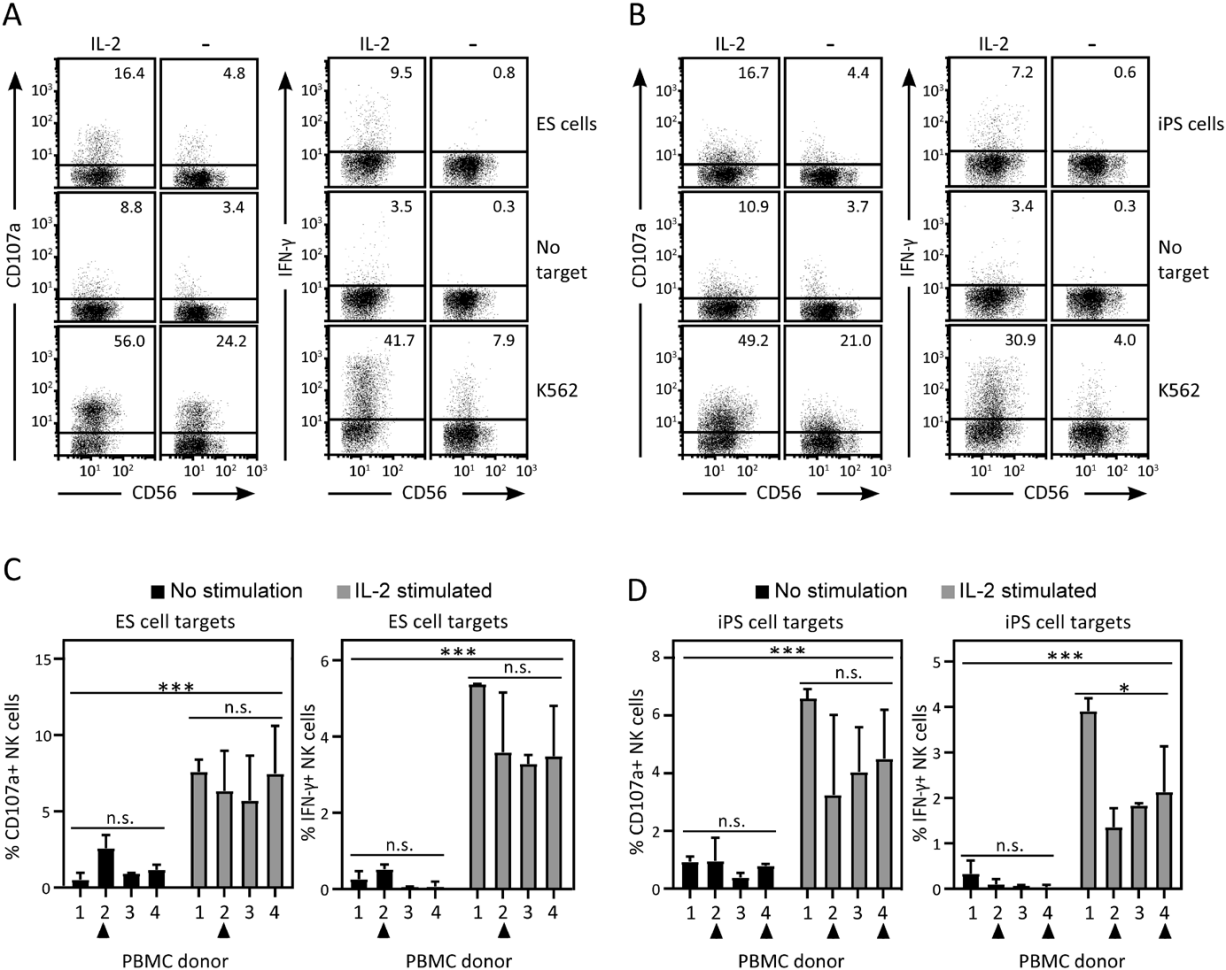
Cytotoxicity and cytokine production by primary NK-cells in response to human pluripotent stem cell targets. Human ES cells (HS360) and an iPS cell line (NCS033) were incubated with blood mononuclear cells from 2 different KIR/HLA matched donors for 4 hours in E8 medium. Both stimulated (incubated with IL-2 overnight) and unstimulated PBMCs (−) were used. Control experiments without target cells or with the NK-susceptible target cell line K562 are shown. (A) NK-cell cytotoxicity (displayed as CD107a surface expression, left panel) and intracellular IFN-γ expression (right panel) in response to co-incubation with ES cells. Flow cytometry dot plots show CD56dim and CD56bright NK cells (gated to exclude CD3+, CD14+, CD19+, CD20+ cells). (B) NK-cell cytotoxicity (left panel) and IFN-γ (right panel) production in response to co-incubation with iPS cells. Percent CD107a+ or IFN-γ+ cells in the displayed experiments are indicated. Data shown are representative of 3 independent experiments. Gating strategy to display NK cells is shown in Supplementary Figure S4. (C) NK-cell cytotoxicity and IFN-γ expression was analyzed as above, using PBMC from 4 different KIR and HLA-typed donors (1-4, Supplementary Table S1). NK-cell/target cell combinations with KIR/HLA mismatch have been indicated with black triangles. Histograms display the fraction of CD107a+ NK cells with the background activation subtracted, error bars display 1 SD, and statistical significance is indicated by asterisks (*P < .05; ***P < .001).

The above findings suggested that pluripotent stem cells are susceptible to NK-cell-mediated killing because they express ligands for the activating receptor NKG2D and lack ligands for inhibitory NK-cell receptors. To investigate whether pluripotent stem cells can be killed by NK cells, we incubated 1 ES cell line (HS360) and 1 iPS cell line (NCS033) with peripheral blood mononuclear cells (PBMC) from healthy donors. Previous papers have demonstrated NK-cell killing of human pluripotent cells, also in a syngeneic setting.^44^ To investigate if NK-cell killing of pluripotent stem cells is increased by KIR/HLA mismatch in alloreactive combinations, we carefully genotyped ES and iPS cell targets and PBMC donors (Supplementary Table S1). Briefly, NK-cells expressing an inhibitory KIR for which they also carry the ligand HLA-C or -B allele will be “educated,” such that they will be inhibited by “self.” These “educated” NK-cells will be able to kill target cells lacking the ligand for that receptor by a missing-self mechanism. Since KIR expression is a stochastic process and subsets of NK cells differ in KIR expression, a KIR/HLA match requires that the target cell carries appropriate HLA-C or -B ligands for all inhibitory KIR responsible for education in the donor. NK-cell cytotoxic activity against pluripotent stem cell targets was assessed by CD107a surface expression on CD3− CD14− CD19− CD20− CD56+ cells following co-incubation with stem cell targets. Whereas only weak CD107a expression could be observed using unstimulated PBMCs, NK-cell cytotoxicity was observed toward both ES and iPS cells using PBMCs cultured with IL-2 overnight (Figure 4A and B). Similarly, co-incubation with both ES and iPS cells induced IFN-γ production in NK cells precultured with IL-2 (Figure 4A and B). The observed level of cytotoxicity was comparable between experiments using KIR/HLA matched and mismatched combinations of PBMC donors and target stem cells (Figure 4C). Together, these observations indicate that ES as well as iPS cells are recognized and killed by NK-cells, and that, importantly, HLA/KIR-matched donor/recipient combinations do not protect pluripotent stem cells from NK-cell-mediated killing.

### Neural progenitor cells differentiated from induced pluripotent stem cells express a ligand for the activating receptor NKp30

**Figure 5.**
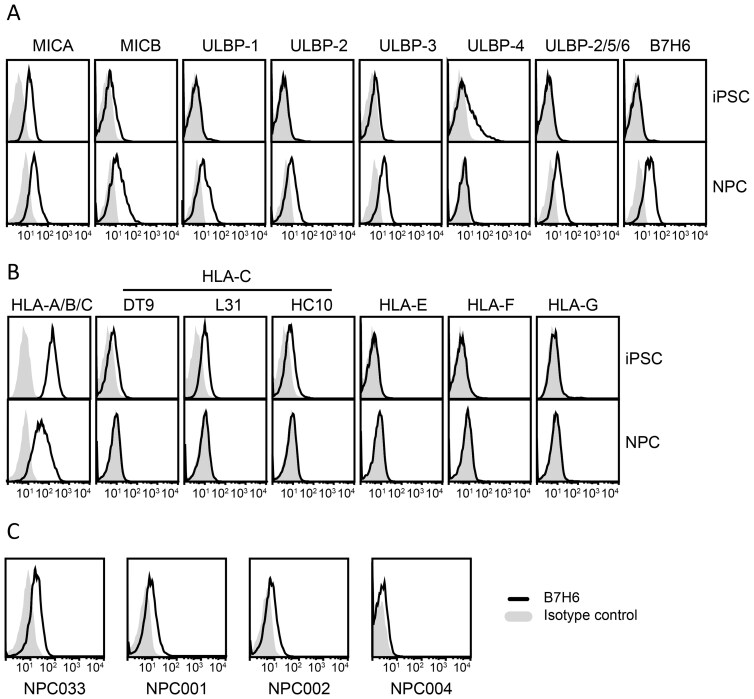
iPS-derived neural progenitor cells exhibit surface expression of the NKp30 ligand B7H6. Neural progenitor cells (NPC) differentiated from iPSC were stained with mAbs against the indicated surface antigens and analyzed by flow cytometry. Data shown are representative of 3 independent experiments. (A) Surface expression of ligands for the activating receptor NKG2D (MICA, MICB, ULBP-1, -2, -3, -4, and -2/5/6) and NKp30 (B7H6) on iPS cell line NCS033 and differentiated NPC033. (B) HLA class I surface expression on NCS033 and NPC033. (C) Histograms displaying surface expression of the NKp30 ligand B7H6 on human NPCs generated from iPSC from NCS033 and 3 additional independent donors. Black lines represent specific mAb staining, control Ig staining is indicated in gray shading.

Clinical applications of pluripotent stem cells involve cells with some level of lineage commitment or differentiation, to minimize the risk of tumorigenesis and to ensure tissue cell-type matching. We therefore wanted to investigate whether the NK-susceptible phenotype of pluripotent stem cells is maintained in more lineage-committed, differentiated cells. For this purpose, we investigated neural progenitor cells (NPCs) derived from iPS cell lines. NPCs showed increased surface expression of the NKG2D ligands MICB, ULBP1, -2, and -3 compared to their iPS cells of origin, whereas ULBP4 expression was decreased (Figure 5A). With regard to HLA-C, very low or no surface expression was observed on NPCs (Figure 7B), comparable to our observation with pluripotent stem cell lines (Figure 2A). Surprisingly, the NKp30 ligand B7H6 was expressed on NPCs (Figure 5A), but not on the corresponding iPS cell line (Figure 1B). B7H6 expression, although at varying levels, was confirmed by flow cytometry analysis of 3 additional NPC populations derived from separate iPS cell donors (Figure 5C). These observations suggest that, in vitro, NPCs are equally or more susceptible to NK-cell-mediated killing than their iPS cells of origin.

### iPS-cell-derived motoneurons express ligands for NKp30 and NKG2D and are killed by NK cells in vitro

**Figure 6.**
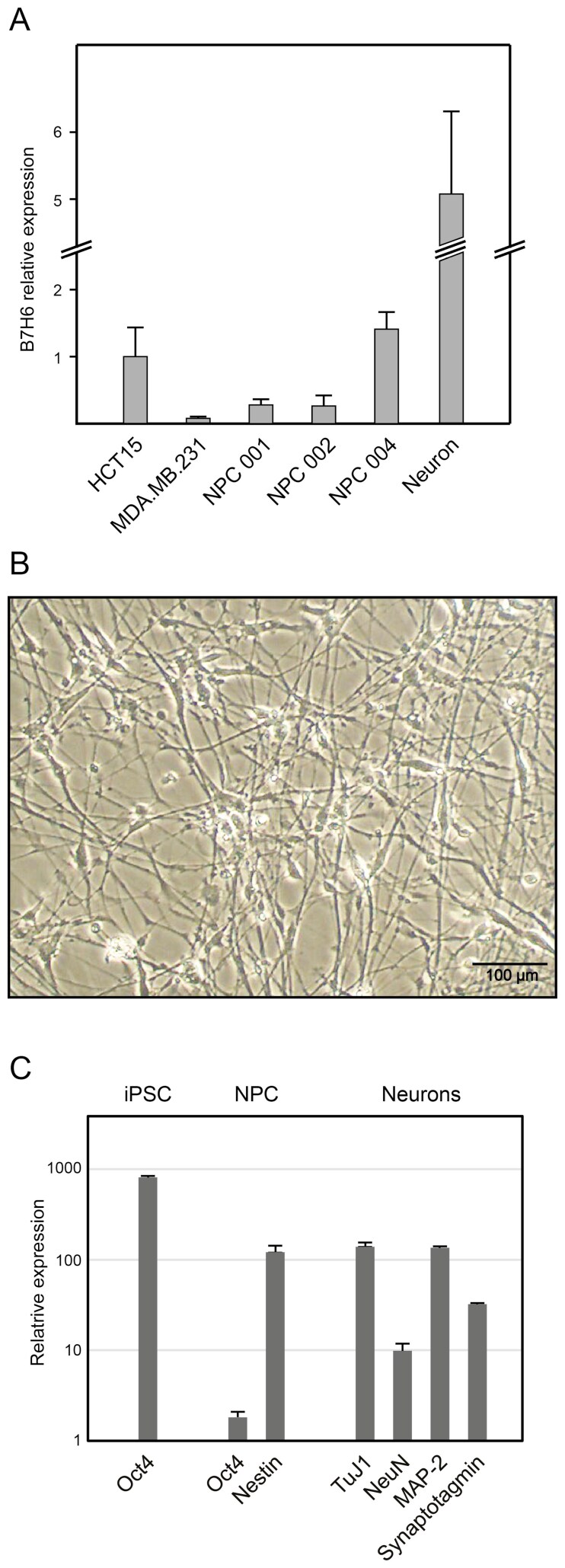
B7H6 expression is maintained in motoneurons differentiated from iPS-derived neural progenitor cells. (A) B7H6 (NCR3LG1) transcription was assessed by qPCR analysis using cDNA from the indicated cells, displaying relative expression using GNB2LI as housekeeping control. The tumor cell lines HCT15 and MDA.MB.231 have been included as B7H6+ and B7H6− controls, respectively. The results are normalized and represent 3 or more independent triplicate experiments. Neurons were generated from iPS-derived NPCs, and their phenotype was documented by (B) phase contrast microscopy demonstrating dendritic morphology, and (C) qPCR analysis for the neuronal markers NeuN (RBFOX3), MAP-2 (MAP2), class III β-tubulin (TuJ1; gene TUBB3), and synaptotagmin (SYT1), as indicated. NPC phenotype was defined by downregulation of the pluripotency marker Oct4 (POU5F1) and concomitant upregulation of nestin (NES). Expression was calculated using β-actin as an internal control. Error bars indicate SD based on 1 triplicate experiment.

MNs were differentiated from iPS-cell-derived NPCs and analyzed by qPCR for the expression of B7H6, alongside the NPCs from 3 different iPS cell lines. Previously characterized tumor cell lines were included as B7H6-positive (HCT15) and B7H6-negative (MDA.MB.231) controls, and GAPDH and GNB2L1 were used as internal control genes. In these experiments, B7H6 (*NCR3LG1*) transcript levels in MNs were high, approximately 5-fold higher compared to the B7H6-positive control cancer cell line HCT15. B7H6 transcription levels in NPCs were lower than in MNs, but significantly higher than in the B7H6-negative control cell line MDA.MB.231 (Figure 6A), corresponding well with the flow cytometry data showing surface expression of B7H6 on all NPCs tested.

**Figure 7.**
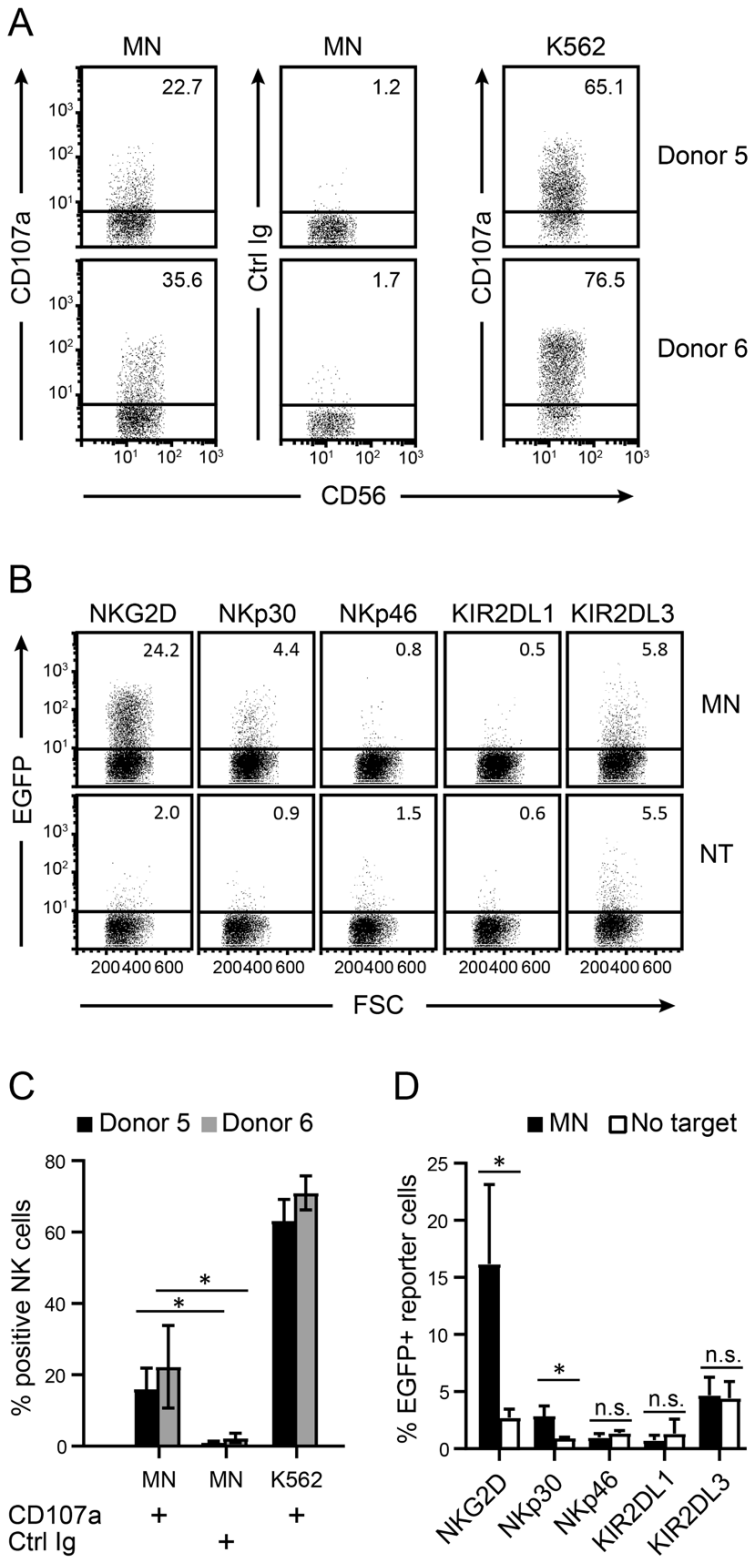
iPS-derived motoneurons trigger NKG2D and B7H6 reporter cells and are killed by primary NK-cells. (A) Motoneurons (MN) differentiated from an iPS cell line were incubated with IL-2-stimulated PBMCs from 2 different donors for 4 hours. The dot plots display % CD107a+ NK cells (gated on CD56+ and CD3−/CD14−/CD19−/CD20− cells) after co-incubation with motoneurons or the K562 cell line as a positive control. Gating strategy is shown in Supplemental Figure S1. (B) Reporter cell assays using reporter cells for the indicated activating or inhibitory receptors. The dot plots display EGFP production by the reporter cells after co-incubation for 6 hours with or without (NT) motoneuron target cells. (C) Histogram with statistics displaying the fraction of CD107a+ NK-cells from 2 different donors after coincubation with motoneurons or K562 cells, compared with isotype Ig control (*n* = 3). Error bars display 1 SD and statistical significance is indicated (*P < .05). (D) Histogram with statistics displaying the fraction of EGFP+ reporter cells for the indicated NK-cell receptors following coincubation with motoneuron target cells or no target cells, *n* = 4 (NKp30), *n* = 3 (NKG2D, KIR2DL1), *n* = 2 (NKp46, KIR2DL2). Error bars display 1 SD and statistical significance is indicated (*:*P* < .03).

Differentiation of NPCs and MNs was verified by morphology, qPCR analysis of lineage-specific differentiation markers, and electrophysiology. MNs developed extensive, branched dendritic trees (Figure 6B). The pluripotency marker Oct4 was downregulated in NPCs concomitant with upregulation of nestin, and both of these markers were supplanted by the differentiation markers NeuN, MAP-2, TuJ1, and synaptotagmin in NPC-derived MNs (Figure 6C). Parallel electrophysiological characterization of separate cultures demonstrated the presence in MNs of overshooting action potentials, voltage-dependent Na+ and K+currents, and *I*-*V* curves characteristic of MNs, as well as spontaneous synaptic currents and potentials (Supplementary Figure S5). To further examine the functional significance of B7H6 expression at the mRNA level, iPS-cell-derived MNs were subjected to in vitro NK-cell cytotoxicity assays. NK-cells from 2 different donors displayed significant cytotoxicity toward MNs (Figure 7A and C), indicating that iPS-cell-derived MNs are efficiently killed by NK-cells in vitro. In reporter cell assays, these MNs reacted strongly with NKG2D reporter cells, and weakly with NKp30 reporters, indicating that ligands for both these receptors are expressed by iPS-cell-derived MNs in vitro (Figure 7B and D). Inversely, reactivity with the inhibitory receptors KIR2DL1 and -2DL3 was not observed.

## Discussion

In this study, we have investigated the extent to which human pluripotent stem cells and cells of the neural lineage differentiated from them engage with specific activating and inhibitory NK-cell receptors, and which corresponding ligands they express on their surface. The results demonstrate that both ES and iPS cells and NPCs and MNs are targets for NK-cell killing. These pluripotent stem cells and derivative neural cells failed to express sufficient levels of the inhibitory ligands HLA-E and HLA-C while expressing several ligands for the activating receptor NKG2D at levels sufficient to trigger NKG2D reporter cells. In accordance, NK-cells showed a cytotoxic response to both types of pluripotent stem cells. In vitro, unstimulated NK-cells responded only weakly, but we observed strong cytotoxic responses after IL-2 stimulation. In vivo, NK-cell responses toward implanted stem cells or stem-cell-derived cells are thus likely to depend on the level of inflammation involved in situ.

NK-cell responses to target cells are regulated by a balance of simultaneous input from activating and inhibitory NK receptors engaged with target cell ligands in a transient immunological synapse.^23^ In this setting, receptor-ligand affinities play a role, together with the local densities of receptors and ligands, and their propensity for clustering in small regions of the membrane to reach a signaling threshold. To dissect the molecular interactions of individual NK-cell receptors with pluripotent stem cell targets, we utilized NK receptor reporter cells. Like NK-cells, these reporter cells form synapses with target cells and thus reflect avidity and local clustering events more closely than assays with soluble receptors, allowing a better assessment of functional sensitivity thresholds. The reporter cell approach is also advantageous because we still lack a complete understanding of the ligands for most activating NK-cell receptors. While the ligands for NKG2D are well characterized, the picture is less clear for the other activating receptors. For NKp30 and NKp46, several alternative ligands have been reported.^39^ The ligand specificities for activating KIRs are still largely unresolved, and several alternative ligands may exist.^28,54^ The basis cell line BWN3G is a modified mouse T-cell line and expresses several other surface receptors that could in theory modulate reporter responses to stem-cell targets. We therefore used several different reporter cell lines that together formed good controls for unspecific activation.

NKG2D ligands have previously been shown to be expressed on cells in different situations of disturbed cellular homeostasis, such as intracellular infection, DNA damage, and cell cycle dysregulation, and are considered surface markers of cellular stress.^55^ Our results here indicate that both ES cells and iPS cells express multiple, partly non-overlapping combinations of NKG2D ligands, in line with previous reports.^41,42^ The important role of NKG2D in NK-cell recognition of pluripotent stem cells was shown in NKG2D (*Klrk1*) deficient mice.^56^ Despite efforts to control media constituents and other physical parameters, in vitro culture can perturb cellular homeostasis. Thus, it remains possible that the expression of NKG2D ligands observed here resulted from stress imposed by the stem cell culture conditions and was not a direct result of pluripotent stem cell transcriptional programs. Nevertheless, such stresses likely apply to all in vitro cultured stem cells, and the observations are therefore highly relevant for potential clinical applications of stem-cell-derived cells.

With regard to ligands for the inhibitory CD94/NKG2A receptor, we did not detect HLA-E expression on any of the ES and iPS cell lines or iPS-derived cells tested. Forced expression of HLA-E on pluripotent stem cells has been reported previously to protect stem cells from NK-cell lysis.^57^ Moreover, only very low levels of surface HLA-C could be detected on ES and iPS cells, functionally unable to trigger reporter cells expressing inhibitory KIR2DL receptors. Although the observed expression of NKG2D ligands could have reflected stem-cell responses to stressful culture conditions, as discussed above, we consider it unlikely that lack of HLA-C and HLA-E expression was a result of stem-cell culture conditions.

In an in vivo teleological perspective, the expression of NKG2D ligands and the lack of expression of HLA-C and -E by pluripotent stem cells would carry the benefit of prohibiting the development of germ-cell-derived embryonal carcinomas or tumors resulting from errors in embryonic implantation and development. NK cells can play a role in the immunological control of tumors with pluripotent origins, such as teratomas.^46,49^ The observed ligand expression by pluripotent stem cells would also mark ectopically located embryo-derived stem cells for killing by NK cells. NK cells are present in the uterine wall before implantation and make up the majority of leukocytes in the decidua during pregnancy. The physiological roles of uterine NK cells are not entirely clear, but important functions appear to be the control of trophoblast invasion and remodeling of spiral arteries. To the extent that inner cell-mass-derived pluripotent cells may erratically escape their trophoblast capsule to invade the maternal tissue, NK cells would be strategically located to detect these cells and kill them. Thus, our observations that ES and iPS cell lines did not express HLA-E and failed to engage and trigger KIR2DL1, -2DL2, and -2DL3 reporters could reflect a transcriptionally programmed feature of pluripotent stem cells, selected through evolution to protect from stem-cell-derived malignancies.

To evaluate to what extent pluripotency is linked to surface expression of ligands for NK-cell receptors, and also to dissect the ability of NK cells to identify and kill teratomas and embryonal carcinomas, 2 human embryonal carcinoma cell lines were assayed with NK-receptor reporter cells. These EC cells largely mirrored the expression profile of ES and iPS cells. Despite the lack of response seen with KIR2DL1, -2DL2, and -2DL3 reporters, flow cytometry experiments suggested some expression of HLA-C, possibly in open conformation. Inhibitory KIR2D receptors bind to the top of HLA-C molecules and form interactions with both α-helices and the presented peptide.^57,58^ Open conformation variants of HLA-C would therefore not be expected to interact with inhibitory KIR receptors. These observations support the idea that the lack of HLA-C and HLA-E expression is tied to a pluripotency transcriptional program.

The concept of cancer stem cells has been the subject of considerable investigation and debate. To the extent that a de-differentiated epigenetic cancer phenotype induces similar surface ligand profiles as found here with pluripotent stem cells, this could contribute to NK-cell-mediated immunosurveillance against malignant cells,^59^ and partly explain the important role of NK cells in prohibiting blood-borne metastasis. Cancer cells are frequently seen to downregulate MHC class I expression, offering escape from killing by cytotoxic T cells.

The NKp30 ligand B7H6 was not expressed by pluripotent stem cells or embryonic carcinoma cell lines but was expressed by iPS-cell-derived NPCs and NPC-derived MNs. Further investigations are necessary to determine if this ligand is temporarily expressed during neuronal development, or also expressed by mature neurons in vivo. The immunogenicity of human stem-cell-derived neuronal progeny is unsettled.^60,61^ Expression of this strong ligand for NKp30 would increase the immunogenicity of neuronal cells derived from pluripotent stem cells. We here found that iPS-derived MNs are targets for NK-cell cytotoxicity in vitro. It is not clear, however, whether these MNs would maintain a susceptible phenotype in vivo. Normal neurons express lower levels of MHC class I than most other cells but have not been reported to express ligands for activating NK-cell receptors in vivo. We are currently investigating mechanisms that regulate B7H6 expression in primary cells as well as cancer cells, aiming also to provide approaches to optimize the expression of this important NK-cell ligand in stem cells and their progeny.

NK cells with cytotoxic capacity can be generated from pluripotent stem cells in vitro,^62^ apparently, fratricide does not present a major problem in these settings. Thus, NK-cell susceptibility does not apply to all differentiated cells generated from pluripotent stem cells in vitro.

NK-cell repertoire development occurs by a stochastic process leading to different receptors being expressed by individual NK cells. In a process termed education, NK cells that by chance lack inhibitory receptors toward HLA-E or self MHC class I alleles become hyporesponsive and will not kill MHC-target cells.^20^ Based on lack of inhibition (missing self), NK-cell alloreactivity is observed in cases where there is an HLA class I allelic mismatch between stem-cell donor and recipient. To the best of our knowledge, none of the previous reports that have shown NK-cell reactivity with pluripotent stem cells have performed KIR and HLA genotyping, and the observed NK-cell responses could thus have been augmented by missing self/NK-cell alloreactivity due to KIR/HLA mismatch between donor and recipient. In our experiments with allogeneic NK cells, combinations of PBMC donors and stem cells were optimized based on HLA-A, -B, and -C and KIR genotyping, considering KIR2DL1, -2DL2/3, and -3DL1 as explained in more detail previously, allowing us to demonstrate that NK-cell responses toward pluripotent stem cells occur even in KIR/HLA matched combinations that mimic the autologous setting. Comparing the NK-cell responses in settings of KIR/HLA match vs mismatch, no significant differences were observed.

Due to HLA identity, one advantage of autologous iPS cells and progenitor and differentiated cells derived from them is the lack of T-cell-mediated transplantation rejection. Due to the time and resources required to generate autologous iPS cells, banks of HLA-homozygous iPS cells would provide a readily available alternative that reduces the problem of T-cell-mediated graft rejection. HLA-C homozygosity (-C1 or -C2 homozygous), however, would in HLA-C1/C2 heterozygous recipients most often render the transplanted cells susceptible to NK-cell killing. A recent strategy using CRISPR-targeted iPS cells that lacked HLA-A and -B but retained 1 HLA-C allele enhanced compatibility with T and NK cells.^16^ However, individual NK cells expressing KIR3DL1 but lacking KIR2DL receptors and CD94/NKG2A would be expected to retain some alloreactive potential toward HLA-B negative transplanted cells. Our in vitro observations here suggest that even autologous or HLA-matched pluripotent stem cells and immature differentiated progeny could induce NK-cell-mediated responses after transplantation, which might limit the clinical effect or lead to a full transplant rejection in the course of a few hours or days.

In a clinical context, the susceptibility of hES and hiPS cells to NK-cell-mediated killing could be harnessed to ensure the elimination of pluripotent stem cells from in vitro populations of differentiated cells intended for therapeutic implantation, thus circumventing the risks of implanting residual pluripotent cells into a patient. That iPS-cell-derived NPCs and MNs are equally susceptible poses the opposite problem: unsuccessful implantation of the intended differentiated cells despite HLA-matching. Potential solutions could include preventing inflammation during implantation to dampen the NK-cell response or modifying the differentiated cells to express inhibitory NK-cell ligands.

## Conclusion

Here, we have demonstrated that pluripotent stem cells and their neural progeny are targets for NK-cell killing both by failing to sufficiently express ligands for inhibitory receptors and by expression of ligands for NKG2D. KIR and HLA-ligand matching did not protect stem cells from N-cell cytotoxicity. Based on our observations we conclude that the surface marker phenotype of therapeutic stem cell derivatives should be carefully considered in order to avoid rejection by NK cells.

## Supplementary Material

sxae083_suppl_Supplementary_Figures

sxae083_suppl_Supplementary_Table

## Data Availability

The data that support the findings of this study are available from the corresponding author upon reasonable request.
