# Supplementary material for: NK-cell cytotoxicity toward pluripotent stem cells and their neural progeny: impacts of activating and inhibitory receptors and KIR/HLA mismatch
Source: Stem Cells. 2024 Dec 21;43(3):sxae083. doi: 10.1093/stmcls/sxae083 (PMC11929945; doi:10.1093/stmcls/sxae083)
Supplement: sxae083_suppl_Supplementary_Figures [file sxae083_suppl_supplementary_figures.zip › Supplementary figures 1-5/Supplementary figures 1-5.docx]

**Supplementary figure 1:** Validation of pluripotency of pluripotent stem cell lines. (**A**) Confocal immunofluorescence microscopy of induced pluripotent stem (iPS) cell line NCS033 stained with antibodies specific for the pluripotency markers Oct4, Nanog and SOX2. Cells were double-stained with Oct4-Cy2 and Nanog-Cy3 and single-stained with SOX2-Cy2. Nuclei were counterstained with DAPI. (**B**) The embryonic stem (ES) cell lines HS360, HS401, HS429 and H9 and the iPS cell line NCS033 were analyzed by quantitative RT-PCR (qPCR) for transcription of the pluripotency markers Oct4 (POU5F1), Nanog (NANOG) and SOX2 (SOX2) compared to β-actin as an internal control. Error bars indicate standard deviation based on one triplicate experiment.

**Supplementary figure 2:** Range of variation in individual reporter cell assays. (**A**) Box plots display reporter cell activation (% EGFP+ reporter cells) after co-incubation with the indicated pluripotent stem cell line targets. n = 3 or more for each reporter cell line. Similarly, (**B**) display variation in reporter cell activation after co-incubation with cell lines NTERA-2 and 2102Ep. Dot plots highlight statistical outliers, n=3 or more for each reporter cell line.

**Supplementary figure 3:** Positive control experiments with reporter cells. Flow cytometry dot plots displaying reporter cell reactivity as EGFP expression. Reporter cells were incubated in plastic wells coated with antibody reactive with the N-terminal FLAG epitope of the chimeric receptors (KIR2DS1, -DS2 and -DS4 and NKp46) or co-incubated with ligand-expressing target cells (721.221 cells transfected with HLA-C*0304 for KIR2DL2 and -2DL3; 721.221 cells transfected with HLA-C*1501 for KIR2DL1; 293T cells naturally expressing the ligands for NKG2D and NKp30). Typical results are shown, percent EGFP-expressing reporter cells are indicated.

**Supplementary figure 4:** Gating strategies for visualizing (**A**) NK cells in cytotoxicity and degranulation assay and (**B**) reporter cells in reporter assays. (**A**) PBMCs were plotted in a SSC/FSC plot and lymphocytes were gated. To separate NK cells from the other lymphocytes, the cells were stained with a FITC-conjugated lineage marker (lin3-FITC) with antibodies for CD3, CD14, CD19 and CD20 (markers for T cells, monocytes and B cells). In addition, the cells were stained with anti-CD56 as an NK cell marker. Lineage marker-negative and CD56-positive cells were gated as shown, and the gated cells were used to show either Pacific Blue-conjugated CD107a antibody binding or PE-conjugated IFN-γ binding. PBMCs incubated without a target cell were used to set the gate. (**B**) The reporter cells (mouse origin) were gated away from the target cells based on their expression of human HLA class I, using the pan-class I antibody W6/32.

**Supplementary figure 5:** Electrophysiological characterization of differentiated MNs. (**A**) Current clamp recordings of repetitive, overshooting action potentials elicited by square wave pulses of increasing current (from top to bottom). (**B**) Voltage clamp recordings of voltage-sensitive inward and outward currents elicited by increasing voltage steps. (**C**) and (**D**) I-V curves for inward Na+ (**C**) and outward K+ (**D**) currents.
