# Supplementary figures and images for: NK-cell cytotoxicity toward pluripotent stem cells and their neural progeny: impacts of activating and inhibitory receptors and KIR/HLA mismatch
Source: Stem Cells. 2024 Dec 21;43(3):sxae083. doi: 10.1093/stmcls/sxae083 (PMC11929945; doi:10.1093/stmcls/sxae083)

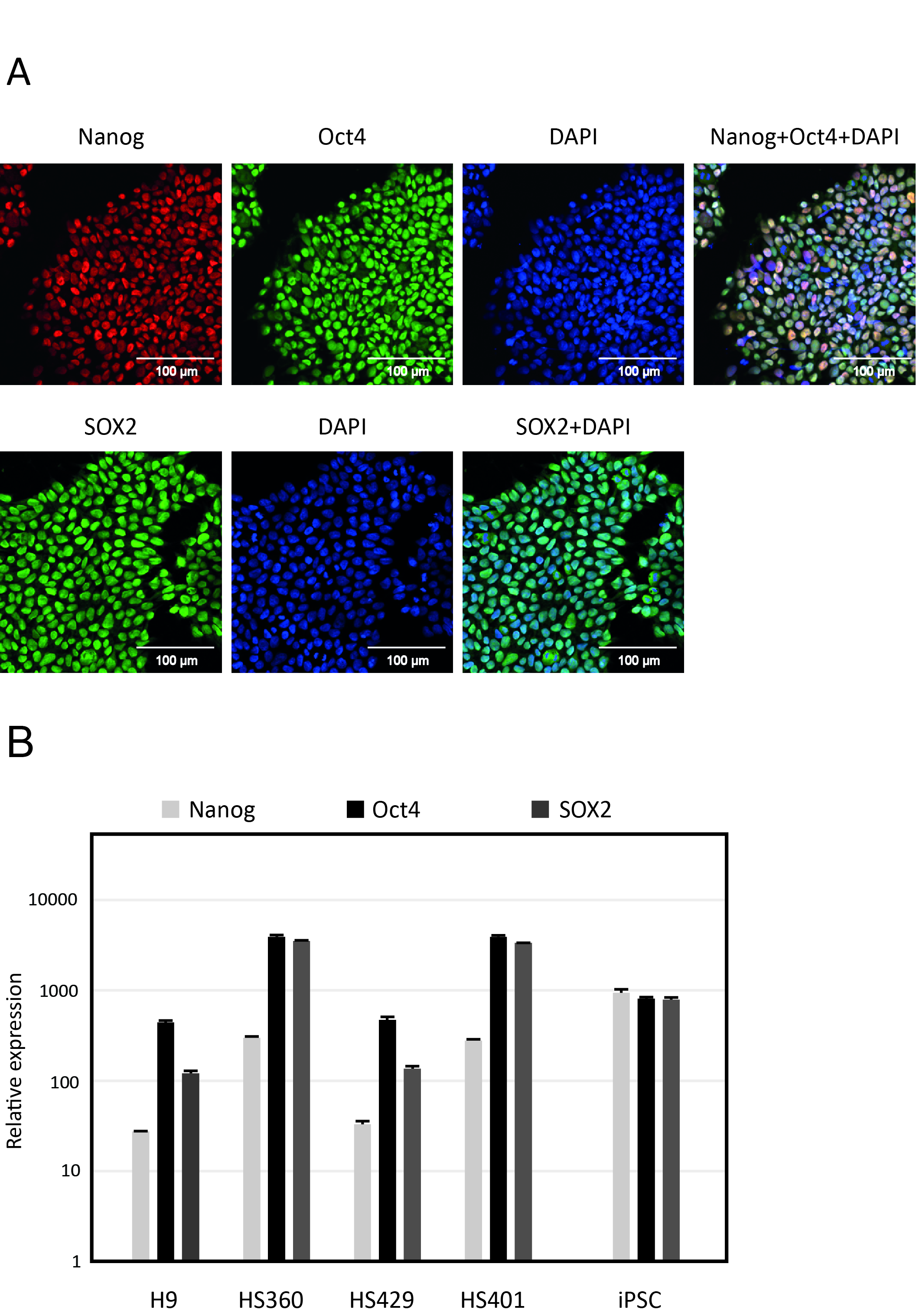

Supplement: sxae083_suppl_Supplementary_Figures [file sxae083_suppl_supplementary_figures.zip › Supplementary figures 1-5/Supplementary Fig 1 ES iPS SB.tif]

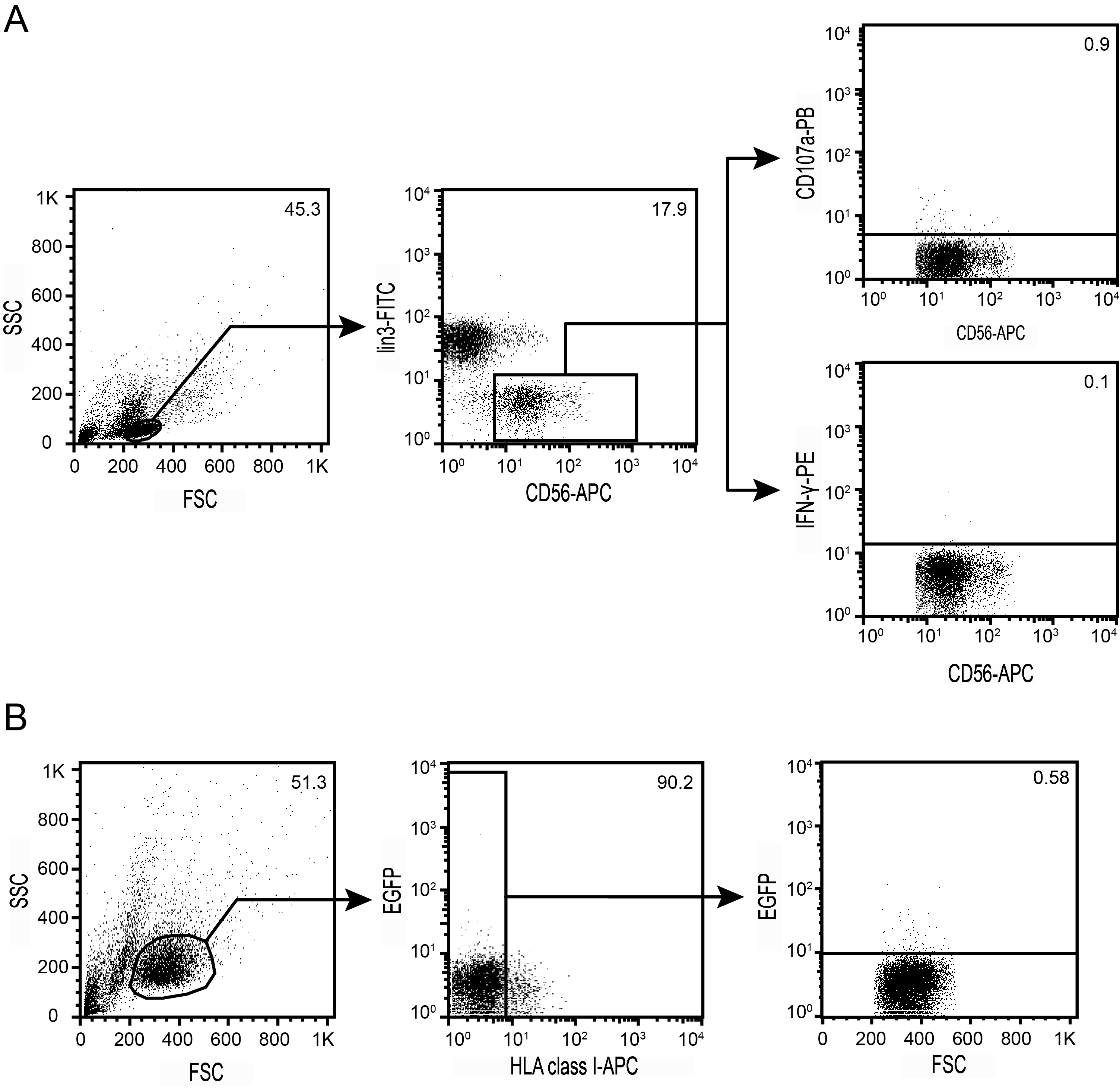

Supplement: sxae083_suppl_Supplementary_Figures [file sxae083_suppl_supplementary_figures.zip › Supplementary figures 1-5/Supplementary fig 4 A+B gating.tif]

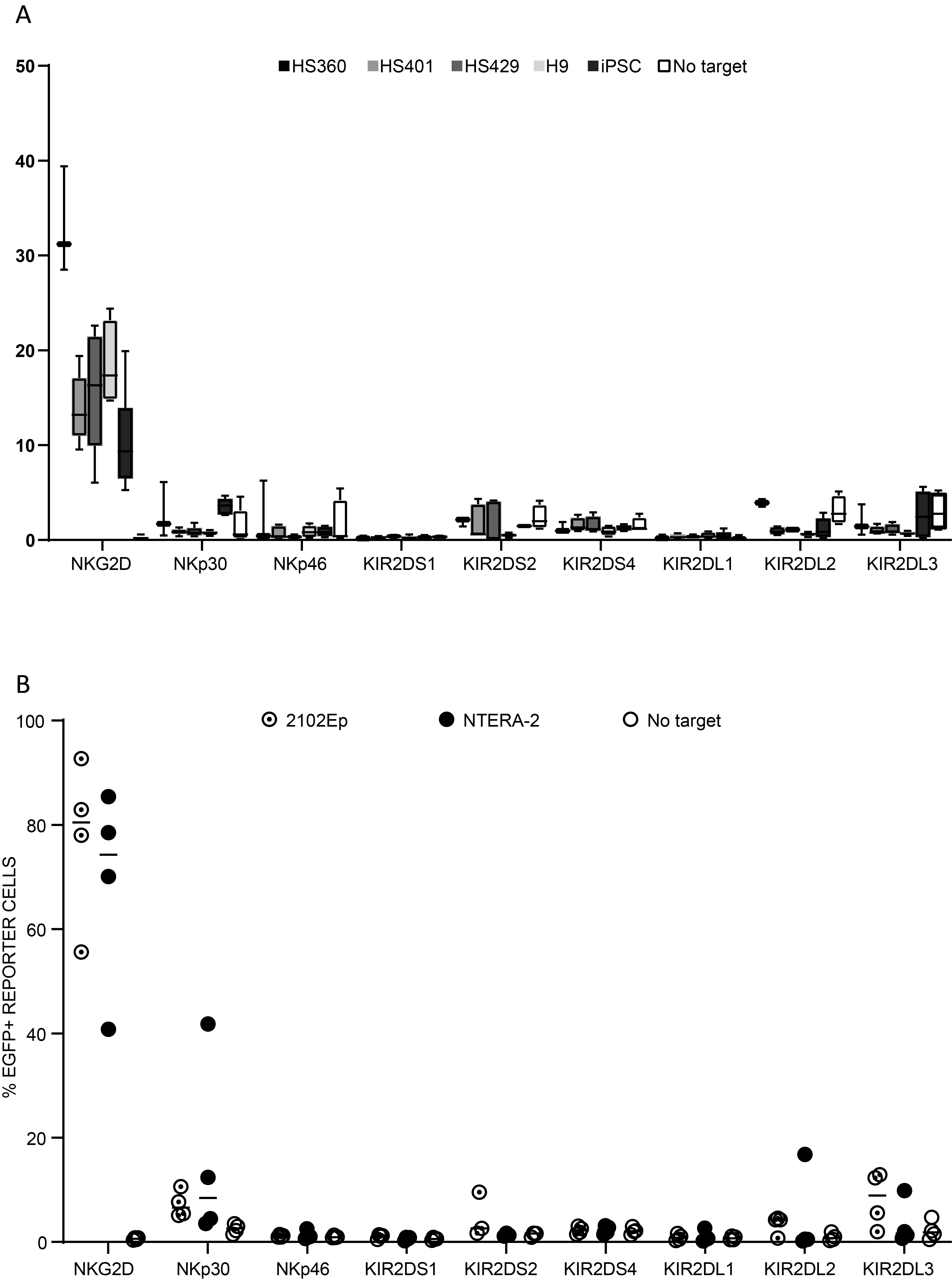

Supplement: sxae083_suppl_Supplementary_Figures [file sxae083_suppl_supplementary_figures.zip › Supplementary figures 1-5/Supplementary figure 2 repA data.tif]

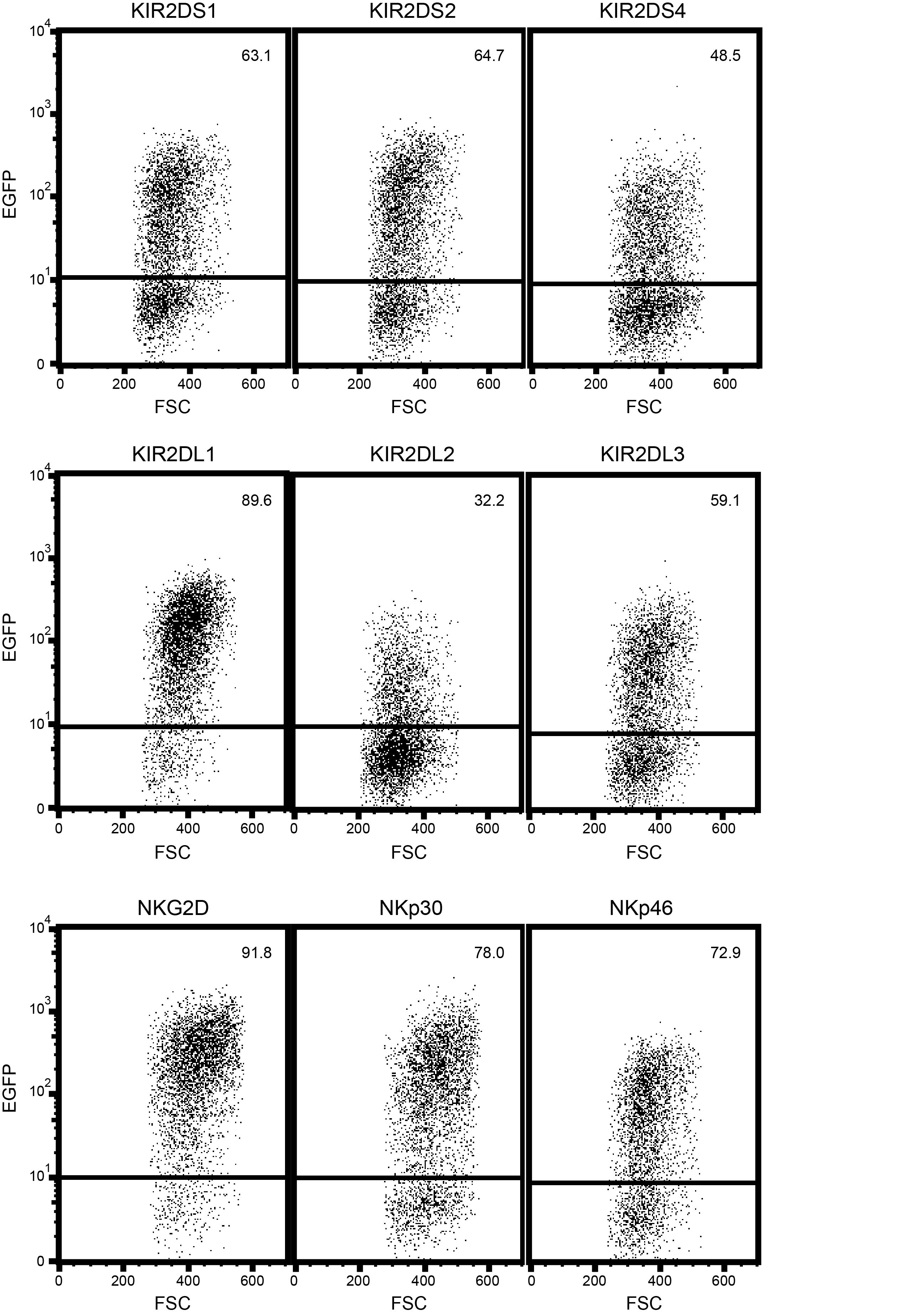

Supplement: sxae083_suppl_Supplementary_Figures [file sxae083_suppl_supplementary_figures.zip › Supplementary figures 1-5/Supplementary figure 3 pos ctrl rep.tif]

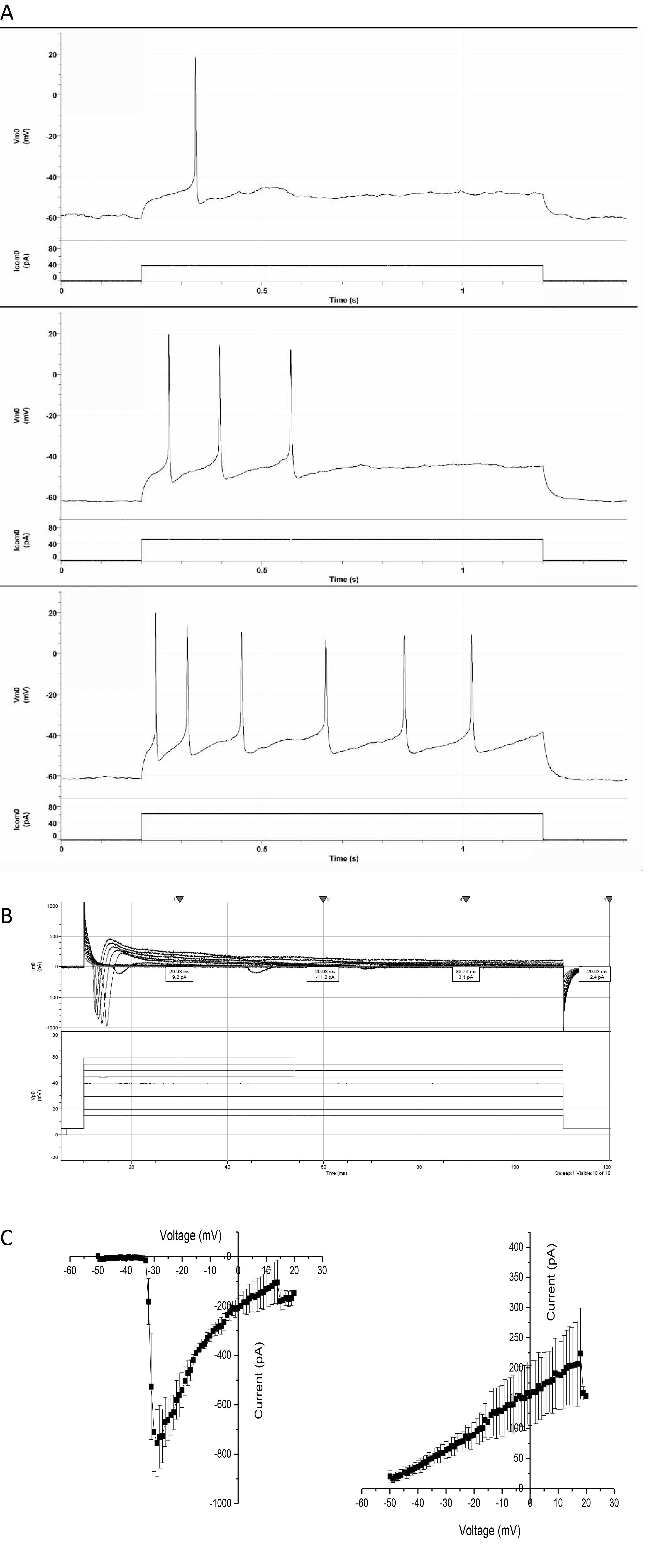

Supplement: sxae083_suppl_Supplementary_Figures [file sxae083_suppl_supplementary_figures.zip › Supplementary figures 1-5/Supplementary figure 5 neuron characterization.tif]
