# Supplementary material for: NK-cell cytotoxicity toward pluripotent stem cells and their neural progeny: impacts of activating and inhibitory receptors and KIR/HLA mismatch
Source: Stem Cells. 2024 Dec 21;43(3):sxae083. doi: 10.1093/stmcls/sxae083 (PMC11929945; doi:10.1093/stmcls/sxae083)
Supplement: sxae083_suppl_Supplementary_Table [file sxae083_suppl_supplementary_table.docx]

**Supplementary table 1:** KIR and HLA class I genotyping of PBMC donors and human pluripotent stem cell lines. Genotyping was achieved using PCR with a sequence specific primer (SSP) typing kit for both KIR and KIR ligand (HLA class I) on extracted DNA from the PBMCs, and KIR ligand on DNA from the stem cell lines.

|  |  | **Receptor** | **Ligand** | **Education** | **HS360** | **NCS033** |
| --- | --- | --- | --- | --- | --- | --- |
| **Donor 1** | KIR2DL1 | yes | no | no |  |  |
|  | KIR2DL2 | yes | C1 Asn80 | **yes** | C1 Asn80 | C1 Asn80 |
|  | KIR2DL3 | yes | C1 Asn80 | **yes** | C1 Asn80 | C1 Asn80 |
|  | KIR3DL1 | yes | no | no |  |  |
| **Donor 2** | KIR2DL1 | yes | C2 Lys80 | **yes** | ***mismatch*** | C2 Lys80 |
|  | KIR2DL2 | yes | C1 Asn80 | **yes** | C1 Asn80 | C1 Asn80 |
|  | KIR2DL3 | yes | C1 Asn80 | **yes** | C1 Asn80 | C1 Asn80 |
|  | KIR3DL1 | yes | B Bw4 | **yes** | B Bw4 | ***mismatch*** |
| **Donor 3** | KIR2DL1 | yes | no | no |  |  |
|  | KIR2DL2 | yes | C1 Asn80 | **yes** | C1 Asn80 | C1 Asn80 |
|  | KIR2DL3 | yes | C1 Asn80 | **yes** | C1 Asn80 | C1 Asn80 |
|  | KIR3DL1 | yes | no | no |  |  |
| **Donor 4** | KIR2DL1 | yes | no | no |  |  |
|  | KIR2DL2 | no | no | no |  |  |
|  | KIR2DL3 | yes | C1 Asn80 | **yes** | C1 Asn80 | C1 Asn80 |
|  | KIR3DL1 | yes | A Bw4 | **yes** | B Bw4 | ***mismatch*** |
